# Hydrocephalus is a rare outcome in community-acquired bacterial meningitis in adults: a retrospective analysis

**DOI:** 10.1186/1471-2334-13-321

**Published:** 2013-07-15

**Authors:** Jacob Bodilsen, Henrik Carl Schønheyder, Henrik Nielsen

**Affiliations:** 1 Department of Infectious Diseases, Aalborg University Hospital, Mølleparkvej 4, PO Box 365, 9100 Aalborg, Denmark; 2 Department of Infectious Diseases, Aalborg University Hospital, Aalborg, Denmark; 3 Department of Clinical Microbiology, Aalborg University Hospital, Aalborg, Denmark

**Keywords:** Hydrocephalus, Bacterial meningitis, Incidence, Cohort studies, Treatment

## Abstract

**Background:**

Community-acquired bacterial meningitis (CABM) continues to have a high mortality rate and often results in severe sequelae among survivors. Lately, an increased effort has been focused on describing the neurological complications of meningitis including hydrocephalus. To aid in this field of research we set out to ascertain the risk and outcome of hydrocephalus in patients with community-acquired bacterial meningitis (CABM) in North Denmark Region.

**Methods:**

We conducted a retrospective population-based cohort study of CABM cases above 14 years of age. Cases diagnosed during a 13-year period, 1998 through 2010, were identified in a laboratory register and data were acquired through patient records. Cases not confirmed by culture met other strict inclusion criteria. The diagnosis of hydrocephalus relied upon the radiologists’ reports on cranial imaging. Outcome was graded according to the Glasgow Outcome Scale at discharge from the primary admission. Long-term sequelae were based upon any subsequent hospital contacts until the end of 2011.

**Results:**

Hydrocephalus was diagnosed in five of 165 episodes (3%) and all were classified as communicating. Only 120 patients had cranial imaging done and in this group the rate was 4.2%. In three cases hydrocephalus was present at admission, while two cases were diagnosed on days 44 and 99, respectively, due to altered mental status. The aetiology was either *Eschericia coli* (n = 2) or *Streptococcus pneumoniae* (n = 3). Case fatality was 60% among cases with hydrocephalus and 17% among other cases. Case fatality was similar irrespective of whether patients had a cranial CT or not.

**Conclusions:**

Hydrocephalus was diagnosed in 3% of adolescent and adult cases with CABM and had a high case fatality rate in spite of specialised medical care and neurosurgical interventions. Our findings are comparable with a recent Dutch national prospective study.

## Background

In recent years several studies have addressed neurological complications in community-acquired bacterial meningitis (CABM) including hydrocephalus [1-3]. Communicating hydrocephalus is the most common form and is due to impaired resorption of CSF through the arachnoid villi [4], while obstructive hydrocephalus is rarely reported [5,6]. A Dutch national prospective cohort study reported a cumulative rate of hydrocephalus of 5% in adult CABM patients with an elevated risk associated with *Listeria monocytogenes*[6]. A retrospective single centre study from Taiwan observed a cumulative rate of hydrocephalus of 21% in a similar setting with *Klebsiella pneumoniae* posing an elevated risk [7]. A systematic review from 2010 analysed the global risk of neurological sequelae from bacterial meningitis in patients older than 1 month and found an overall rate of hydrocephalus of 7.1% [8]. Of note, four in five patients included were below five years of age. Hydrocephalus is most likely associated with an increased case fatality [3,6,7] and a clearer picture of the frequency and risk of this complication could be conducive to improvement of therapy. Therefore, we have conducted a retrospective population-based study of the occurrence, risk and outcome of hydrocephalus in CABM in North Denmark Region from 1998 through 2010.

## Methods

### Setting and study population

We conducted a retrospective cohort study on patients with CABM from Jan. 1^st^ 1998 to Dec. 31^st^ 2010 in North Denmark Region (formerly North Jutland County), which had a population of 493.298 in 1998, and 579.628 inhabitants in 2010. The increase in the size of the catchment population was due to an administrative reform in Denmark in 2007.

Throughout the study period, the region was served by six district hospitals and one university hospital, Aalborg University Hospital, at which the Department of Clinical Microbiology provided diagnostic services to the entire population throughout the study period.

In Denmark, all hospital care is tax-paid and thus free of charge. A unique personal identification number is assigned to all residents by which all information on hospital associated care was extracted from registries and patient records.

Cases of CABM were retrieved from the laboratory information system (ADBakt, Autonik, Sweden) in the Department of Clinical Microbiology, Aalborg Hospital.

Patients were eligible if they were older than 14 years of age, had a clinical picture strongly suggesting CABM (headache, fever, stiffness of the neck, petechiae, confusion or impaired level of consciousness) and ≥1 of the following inclusion criteria were fulfilled:

1. Positive CSF culture.

2. Positive blood culture and one or more of the following CSF findings: >10 leukocytes/mL [9]; glucose index <0.23; CSF glucose <1.9 mmol/L; protein >2.2 g/L [10].

3. Presence of bacteria in Gram stain of CSF.

4. Non-culture detection of bacteria in CSF by either gene amplification or antigen test.

If a patient fulfilled multiple criteria only the strongest criterion was noted (1 > 2 > 3 > 4).

Exclusion criteria were:

1. Cerebral abscess.

2. Hospital-acquired bacterial meningitis as defined by the Centers of Disease Control [11].

3. Known hydrocephalus or implanted neurosurgical device.

4. The clinical record could not be retrieved (n = 2).

### Patient data

Clinical, radiological and laboratory data were retrieved from hospital records, including records from hospitals in other regions if the patient had been transferred. Three patients were foreign tourists and therefore data only exists from the primary admission.

A regional guideline for the treatment of CABM in adults recommended penicillin G as monotherapy with the addition of gentamicin in elderly patients. In 2003, the guideline was amended with dexamethasone treatment preferably before and not later than one hour after administration of intravenous antibiotics [12]. In 2009 the recommended antibiotic regimen was changed to penicillin G and cefotaxime [13].

A diagnosis of hydrocephalus was based on the radiologist’s written report of cranial imaging while the patient had meningitis or at other subsequent admissions (follow-up ended with completion of the record review in 2011). Evans’ index (a linear ratio between the maximal frontal horn width and the maximal width of the inner skull - normally <0.3 [14]) was calculated when the radiologist’s report suggested hydrocephalus whenever possible. There were no routine follow-up CT-scans of patients with meningitis.

Outcome was graded according to the Glasgow Outcome Scale (GOS) based on hospital records at discharge from the primary admission. A GOS of 1–4 was considered an unfavourable outcome, while a GOS of 5 was considered a favourable outcome. Long-term sequelae were based upon the primary admission and any subsequent admissions or out-patient hospital contacts within the region. Patients admitted or transferred to the Department of Infectious Diseases had a scheduled clinical follow-up 1–3 months after discharge.

Length of hospital stay was calculated until discharge alive, in-hospital death or transfer to a rehabilitation unit.

The study was notified to the North Denmark Region in accordance with a directive from the Danish Data Protection Agency; permission from the health research ethics committee was not required. For further inquiry please see http://www.datatilsynet.dk/english.

### Statistics

Categorical variables were analysed using Fisher’s exact test. A two-tailed p-value <0.05 was considered significant.

## Results

During the study period we identified 165 cases of CABM, five (3.0%) of whom developed hydrocephalus. In the subgroup of 120 patients with cranial imaging done during admission (114 initially and six in the course of admission), the rate was 4.2%. Evans’ index was 0.39 and 0.43 in the two instances in which CT-images had been saved. Two of the patients with hydrocephalus also had ischaemic cerebral infarctions during admission (one before development of hydrocephalus and one after the diagnosis of hydrocephalus). A third patient had had an ischaemic cerebral infarction 2.5 months prior to admission for CABM with hydrocephalus and a fourth patient had a haemorrhagic infarction during admission after the diagnosis of hydrocephalus.

The bacterial aetiology is listed in Table 1, while the demographic and clinical characteristics are presented in Table 2. The patients were 40 to 77 years of age (three females, two males). One had rheumatoid arthritis and was treated with methotrexate and corticosteroids. Another patient with a previous incident of meningococcal meningitis had no known immunodeficiency and had had two previous CT scans without any pathological findings before the index CABM. A third patient had a diagnosis of lipomas in the right frontal lobe and Sylvian fissure without any progression on MRI five and two years before the current episode of CABM and hydrocephalus. The remaining two patients had no comorbidities.

**Table 1:** Bacterial aetiology of community-acquired meningitis in North Denmark Region 1998-2010

**Bacteriology:**	**Total**
***Streptococcus pneumoniae***^***a***^	83
***Neisseria meningitidis***	47
**Haemolytic streptococci groups A, B and G**	9
***Staphylococcus aureus***	7
***Eschericia coli***^***a***^	6
***Listeria monocytogenes***	4
**Non-haemolytic streptococci**	2
***Haemophilus influenza***	2
***Enterococcus faecalis***	2
***Pasteurella multocida***	1
***Capnocytophaga canimorsus***	1
**Gram positive cocci, species diagnosis not obtained**	1
**Sum:**	165

148 were diagnosed by inclusion criterion 1, seven by inclusion criterion 2, eight by inclusion criterion 3 and two by inclusion criterion 4.

^a^ The bacterial aetiologies in the five cases with hydrocephalus were *S. pneumoniae* (3) and *E. coli* (2).

**Table 2:** Clinical and laboratory features of bacterial meningitis patients with and without hydrocephalus

**Characteristics**^**a**^	**Patients with hydrocephalus**	**Scanned patients without hydrocephalus**
	**(n = 5)**	**(n = 115)**
**Symptom duration (days)**	3.5	2
**Headache**	3	66
**Nausea**	2	54
**Confusion**	4	114
**GCS**^**b**^		
<9	1	19
9-12	1	49
>12	3	47
**MAP (mm Hg)**	104	N/A
**ICP (mm Hg)**	-	
**Heart rate (beats/min)**	85	100
**CRP (mg/L)**	293	232
**Cranial nerve paresis**	2	22
**Sensory / motor nerve deficit**	0	11
**Seizures before/during admission**	0	14
**CSF leukocytes (x10^**^**6**^**/L)**	2766	1702
**Polymorphonuclear leukocytes (%)**	84	92
**CSF erythrocytes (x10^**^**6**^**/L)**	115	140
**CSF:blood glucose index**	0.005	4
**CSF protein (g/L)**	5.9	455
**Positive CSF culture**	5	103
**Positive blood culture**	2	86
**Antibiotics**		
Day 1	4	99
Day 2	0	9
After day 2	1	7^c^
**Dexamethasone**^**d**^	0	30
**GOS**^**e**^		
Unfavourable outcome (GOS 1–4)	5	55
Favourable outcome (GOS 5)	0	60
**Length of stay (days)**	17 (3–51)	19 (1–82)

^a^ Median values are used for continuous variables.

^b^ Not all patients had Glasgow Coma Scale score estimated in the records at admission but could be assessed to have a score <9, 9–12 or >12 based upon data review.

^c^ One patient never scanned was not given appropriate antibiotic therapy during admission for unclear reasons.

^d^ 10 mg of dexamethasone q.i.d. for four days initiated preferably before and not later than within one hour of administration of intravenous antibiotics.

^e^ Glasgow Outcome Scale: 1. Death, 2. Vegetative state, 3. Dependent upon others in daily life, 4. Sequelae, but independent life, 5. No or only minor sequelae.

Abbreviations: *CSF* Cerebrospinal fluid, *GCS* Glasgow coma scale, *GOS* Glasgow outcome scale, *ICP* Intracranial pressure, *MAP* Mean arterial pressure, *N/A* Not available.

Intracranial pressures (ICP) were measured initially in two of the five patients with values recorded as 15 mm Hg (with subsequent rising pressure) and 70 mm Hg, respectively. The first patient was treated with a bundle of interventions including deep sedation, hypertonic intravenous fluids, elevated bedrest and vasopressors. An extraventricular drainage was inserted but eventually cerebellar herniation occurred on the eighth day of admission. The same bundle of interventions were applied for the second patient and supplemented with hypothermia. The patient survived and was diagnosed with hydrocephalus on day 44 because of altered mental status. In a third patient, who had no measurements of ICP from the time of admission for CABM, hydrocephalus was diagnosed on day 99 also due to an altered mental status. Likewise, ICP measurements were not obtained in the last two cases with hydrocephalus present at admission. Most probably a dismal prognosis was anticipated due to significant comorbidities and intensive care and resuscitation were waived (one of the patients died at a hospice on the day of discharge).

Hydrocephalus occurred in two cases of CABM caused by *Escherichia coli*, but in none of our four cases with *L. monocytogenes*. Of note, one of the patients with *L. monocytogenes* later developed a post-infectious syringomyelitis.

Four of the patients with hydrocephalus received immediate appropriate antibiotic therapy on the day of admission, whereas optimal treatment was delayed for 36 hours in the last patient who had an indistinctive clinical presentation. There was no difference in the median time to administration of appropriate antibiotics between patients with or without hydrocephalus. A total of 88 patients were included after 2003, when dexamethasone was implemented into the treatment guidelines, of which 39 (44%) received dexamethasone appropriately. None of the patients with hydrocephalus received dexamethasone appropriately even though three cases occurred after 2003. The median length of hospital stay among patients with hydrocephalus was 17 days.

Case fatality and GOS scores of patients stratified by presence of hydrocephalus or not and by performed CT scan or not are shown in Table 3. Case fatality was not significantly different among scanned and non-scanned patients. However, patients who were scanned were more likely to have an unfavourable outcome than patients who were never scanned (50% vs. 22%, p = 0.04). All in all, three of five patients with hydrocephalus had a fatal outcome and all deaths were ascribed to herniation/neurological causes. The two surviving patients with hydrocephalus diagnosed on day 99 and 44, respectively, had normal intracranial pressures at subsequent lumbar punctures and hence did not require surgical intervention. They did, however, have considerable long-term sequelae ranging from hearing deficits, headache, and motor/sensory nerve deficits to neuropsychologically verified concentration difficulties and memory loss leading to an early retirement. One of them also had septic arthritis during the course of admission for CABM with the same causative pathogen (*E. coli*).

**Table 3:** Outcome of patients with bacterial meningitis in North Denmark Region 1998–2010

	**Unfavourable outcome (GOS**^**a **^**1–4)**
**Meningitis complicated with hydrocephalus (n = 5)**	5/5^b^
**Meningitis without hydrocephalus (n = 160)**	65/160
**Meningitis and cranial CT scan done (n = 120)**	60/120^c^
**Meningitis and cranial CT scan not done (n = 45)**	10^d^/45

^a^ Glasgow outcome scale: 1. Death, 2. Vegetative state, 3. Dependent upon others in daily life, 4. Sequelae, but independent life, 5. No or only minor sequelae.

^b^ Two patients also had ischaemic cerebral infarctions (one before hydrocephalus, one after the diagnosis of hydrocephalus). Another patient with known lipomas in the right frontal lobe and the Sylvian fissure also had small haemorrhages at and during admission. One additional patient had been admitted 2.5 months earlier with cerebral infarction.

^c^ The following intracranial abnormalities were found by cerebral CT scans in patients with an unfavourable outcome (including the five patients with hydrocephalus): new cerebral ischaemic infarctions 16, haemorrhagic cerebral infarctions 7, sinus thrombosis 1, benign cerebral tumours 5 (1 lipoma, 4 meningioma), septic emboli 2, ventriculitis 1, hygroma 1, internal carotid artery aneurysm 1, ventricular empyema 1, chronic subdural haematoma 1, basal skull fracture 1, epidural absces (C2-C3) 1, cerebral abscess 1 (developed day 31). Some patients had more than one abnormal finding.

^d^ A total of eight patients died in the group never scanned. In seven patients circulatory instability precluded scanning and all died within the first two days of admission.

## Discussion

This retrospective Danish population-based study of hydrocephalus complicating CABM found a risk of hydrocephalus comparable with a recent Dutch national prospective study [6]. Taking the 120 patients with cranial imaging as denominator, the incidence of hydrocephalus was 4.2%, which is notably lower than in multiple older and even some recent studies [1,2,6-8,15,16]. Interestingly, we found two cases of hydrocephalus occurring within three months after the incident episode of meningitis. None of the cases that developed hydrocephalus had been treated with dexamethasone.

We could not confirm previous associations between either *L. monocytogenes* or *K. pneumoniae* meningitis and hydrocephalus, but it should be noticed that two cases were caused by *E. coli*, which belong to the same family as *K. pneumoniae*, the Enterobacteriaceae. Our study confirmed a high case fatality in CABM patients with hydrocephalus [6,7]. We cannot rule out potential confounders regarding case fatality and CABM with hydrocephalus. The long-term sequelae in the two survivors with hydrocephalus were considered unrelated to hydrocephalus as lumbar punctures after diagnosis had revealed normal pressures.

Our study had both significant strengths and limitations. The health care system with universal coverage and the use of a unique personal identifier for all health care contacts limited selection bias and secured long-term follow-up. However, significant information bias must be reckoned with as only a subset of patients were scanned (some were circulatory unstable and rapidly deteriorated to a fatal outcome) and not all patients scanned initially were rescanned if they descended in level of consciousness. This may have resulted in an underestimation of the incidence of hydrocephalus which was actually lower than in some studies where all patients had undergone cranial imaging [1,2] thereby also increasing numbers of pathological findings, some of which may have been incidental. The long period of follow-up makes it unlikely that cases with symptomatic progressive hydrocephalus should have been missed. Furthermore, patients never scanned had the same case fatality (8/45, 17.8%) as the entire study group. It is also noteworthy that a significantly larger proportion of patients never scanned had a favourable outcome with no or only minor sequelae and fewer, if any, long-term sequelae. The higher morbidity among scanned patients is probably a result of confounding by indication.

It should also be considered that some patients with CABM (and potentially hydrocephalus) presented with septic shock and were given antibiotics before a lumbar puncture was performed. This would also be a limitation to other studies of bacterial meningitis and it was probably not important in our study due to the inclusion of patients fulfilling additional criteria in case of a negative CSF culture.

The low risk of hydrocephalus meant a lack of power to identify predictors among the multiple variables examined. Still, we noticed that neither Glasgow Coma Scale score at admission, duration of symptoms before admission, time to administration of first adequate dose of antibiotic nor lack of administration of dexamethasone were associated with hydrocephalus. Despite being an infrequent complication in patients with CABM, the clinicians must be familiar with it and its prognostic significance in order to initiate diagnostic measures and adequate and timely treatment.

## Conclusion

During a 13-year period the cumulative incidence of hydrocephalus in community-acquired bacterial meningitis was 3% in North Denmark Region. However, the incidence may be underestimated as eight patients, who were never scanned, died. The limited number of cases did not allow identification of clinical correlates and risk factors for hydrocephalus. Despite being a rare agent*, E. coli* accounted for two of five cases and thus showed a risk profile similar to that described for *K. pneumoniae*. The high case fatality rate associated with hydrocephalus in CABM patients emphasises the need for further studies of the pathophysiological mechanisms and effective treatment. This calls for multi-centre cooperation in future studies.

## Abbreviations

CABM: Community-acquired bacterial meningitis; CSF: Cerebrospinal fluid; GCS: Glasgow coma scale; GOS: Glasgow outcome scale; ICP: Intracranial pressure; MAP: Mean arterial pressure; N/A: Not available; NA: Noradrenaline

## Competing interests

The authors declare that they have no competing interests.

## Authors’ contributions

JB collected all the data, analysed and interpreted the information and wrote the 1st draft of the manuscript. HCS assisted in data analysis, interpretation, writing and proof-reading of the manuscript. HN initialised the study and helped in design, data analysis, interpretation, writing and proof-reading of the manuscript. All authors’ read and approved the final manuscript.

## Pre-publication history

The pre-publication history for this paper can be accessed here:

http://www.biomedcentral.com/1471-2334/13/321/prepub
